# Sample size calculation for microarray experiments with blocked one-way design

**DOI:** 10.1186/1471-2105-10-164

**Published:** 2009-05-28

**Authors:** Sin-Ho Jung, Insuk Sohn, Stephen L George, Liping Feng, Phyllis C Leppert

**Affiliations:** 1 Department of Biostatistics and Bioinformatics, Duke University Medical Center, North Carolina 27710, USA; 2 Center for Fibroid Biology and Therapy, Duke University Medical Center, North Carolina 27710, USA

## Abstract

**Background:**

One of the main objectives of microarray analysis is to identify differentially expressed genes for different types of cells or treatments. Many statistical methods have been proposed to assess the treatment effects in microarray experiments.

**Results:**

In this paper, we consider discovery of the genes that are differentially expressed among *K *(> 2) treatments when each set of *K *arrays consists of a block. In this case, the array data among *K *treatments tend to be correlated because of block effect. We propose to use the blocked one-way ANOVA *F*-statistic to test if each gene is differentially expressed among *K *treatments. The marginal p-values are calculated using a permutation method accounting for the block effect, adjusting for the multiplicity of the testing procedure by controlling the false discovery rate (FDR). We propose a sample size calculation method for microarray experiments with a blocked one-way design. With FDR level and effect sizes of genes specified, our formula provides a sample size for a given number of true discoveries.

**Conclusion:**

The calculated sample size is shown via simulations to provide an accurate number of true discoveries while controlling the FDR at the desired level.

## Background

Clinical and translational medicine have benefited from genome-wide expression profiling across two or more independent samples, such as various diseased tissues compared to normal tissue. DNA microarray is a high throughput biotechnology designed to measure simultaneously the expression level of tens of thousands of genes in cells. Microarray studies provide the means to understand the mechanisms of disease. However, various sources of error can influence microarray results [1]. Microarrays also present unique statistical problems because the data are high dimensional and are insufficiently replicated in many instances. Methods of adjustment for multiple testing therefore become extremely important. Multiple testing methods controlling the false discovery rate (FDR) [2] have been popularly used because they are easy to calculate and less strict in controlling the false positivity compared to the family-wise error rate (FWER) control method [3].

Numerous sample size calculation methods have been proposed for comparing *independent *groups while controlling the FDR in designing microarray studies. Lee and Whitmore [4] considered comparing multiple groups using ANOVA models and derived the relationship between the effect sizes and the FDR using a Bayesian approach. Their power analysis does not address the multiple testing issue. Muller *et al*. [5] chose a pair of testing errors, including FDR, and minimized one while controlling the other at a specified level using a Bayesian decision rule. Jung [6] proposed a closed form sample size formula for a specified number of true rejections while controlling the FDR at a desired level. Pounds and Cheng [7] and Liu and Hwang [8] proposed similar sample size formulas which can be used for comparison of *K *independent samples. These methods are for the FDR-control methods based on independence or a weak dependency assumption among test statistics. Recently, Shao and Tseng [9] introduced an approach for calculating sample sizes for multiple comparisons accounting for dependency among test statistics.

In some studies, specimens for *K *treatments are collected from the same subject and means are compared across treatment groups. In this case, the gene expression data for the *K *treatments may be dependent since they share the same physiological conditions. For example, Feng *et al*. [10] conducted a study to discover the genes differentially expressed between center (C) and edge (E) of the uterine fibroid and the matched adjacent myometrium (M). In this study, specimens are taken from the three sites for each patient. The patients are blocks and the three sites (*K *= 3), C, E and M, are treatments (or groups) to be compared.

Since a set of *K *specimens are collected from each patient, we require a much smaller number of patients than a regular unblocked design. Furthermore, the observations within each block tend to be positively correlated, so that a blocked design requires a smaller number of arrays than the corresponding unblocked design just as a paired two-sample design with a positive pairwise correlation requires a smaller number of observations than a two independent sample design. The more heterogeneous the blocks are, the greater the savings in number of arrays for the blocked design.

In this paper, we consider a non-parametric blocked *F*-test statistic to compare the gene expression level among *K *dependent groups. We adjust for multiple testing and control the FDR by employing a permutation method. We propose a sample size calculation method for a specified number of true rejections while controlling the FDR at a specified level. Through simulations, we show that the blocked *F*-test accurately controls the FDR using the permutation resampling method and the calculated sample size provides an accurate number of true rejections while controlling the FDR at the desired level. For illustration, the proposed methods are applied to the fibroid study [10] mentioned above.

## Methods

### Non-parametric block *F*-test statistic

Suppose that we want to discover genes that are differentially expressed among *K *sites (treatments or groups). For each of *n *patients (blocks), a specimen is collected from each site for a microarray experiment on *m *genes. In this case, the gene expression data from the *K *sites tend to be correlated. Let *Y*_*ijk *_denote the expression level of gene *i *(= 1,..., *m*) from treatment *k *(= 1,..., *K*) of block *j *(= 1,..., *n*). We consider the blocked one-way ANOVA model

(1)

where, for gene *i*, *μ*_*i *_is the population mean, *δ*_*ik *_is a fixed treatment effect and the primary interest, *γ*_*ij *_is a random block effect, and *ε*_*ijk *_is a random error term. We assume that , *γ*_*i*1_,..., *γ*_*in *_are independent and identically distributed (IID) with mean 0 and variance *v*_*i*_, (*ε*_*ijk*_, 1 ≤ *j *≤ *n*, 1 ≤ *k *≤ *K*) are IID with mean 0 and variance , and error terms and block effects are independent. The standard ANOVA theory using parametric *F *distributions to test the treatment effect assumes a normal distribution for *ε*_*ijk*_. However, in this paper, we avoid the normality assumption by using a permutation resampling method in testing and a large-sample approximation in sample size calculation.

For gene *i*(= 1,..., *m*), the hypotheses for testing the treatment effect are described as



against



We reject *H*_*i *_in favor of  for a large value of *F*-test statistic

(2)

where , and . If the error terms are normally distributed, *F*_*i *_marginally has the *F*_*K*-1, (*K*-1)(*n*-1) _distribution under *H*_*i*_. The normality assumption can be relaxed if *n *is large.

Without the normality assumption, the joint null distribution of the statistics can be approximated using a block permutation method, where the array data sets for *K *treatments are randomly shuffled within each block: the permuted data may be represented as , where  is a random permutation of (1,..., *K*). Note that there are (*K*!)^*n *^different permutations, among which (*K*!)^*n*-1 ^give different *F*-statistic values. The R language package multtest [11] can be used to implement the permutation-based multiple testing procedure for blocked microarray data. We consider adjusting for the multiplicity of the testing procedure by controlling the FDR [12,13].

#### Permutation-based multiple testing for FDR-control

(i) Compute the *F*-test statistics (*F*_1_,..., *F*_*m*_) from the original data, (*f*_1_,..., *f*_*m*_).

(ii) From the *b*-th permutation data (*b *= 1,..., *B*), compute the *F*-test statistics .

(iii) For gene *i*, estimate the marginal p-value by



where *I*(*A*) is an indicator function of event *A*.

(iv) For a chosen constant *λ *∈ (0, 1), estimate the q-value by



(v) For a specified FDR level q*, discover gene *i *(or reject *H*_*i*_) if *q*_*i*_ < q*.

### Sample size calculation

Let ℳ_0 _and ℳ_1 _denote the sets of indices of genes that are equally and differentially expressed, respectively, in *K *treatments, and { = *δ*_*ik*_/*σ*_*i*_, *i *∈ ℳ_1_, 1 ≤ *k *≤ *K*} denote the standardized effect sizes for the differentially expressed genes. Let *m*_0 _and *m*_1 _= *m *- *m*_0 _denote the cardinalities of ℳ_0 _and ℳ_1_, respectively.

Suppose that we want to discover gene *i *(or reject *H*_*i*_) if the marginal p-value *p*_*i *_is smaller than *α *∈ (0, 1). For large *m *and under the independence assumption or weak dependence among the *F*-test statistics, the FDR corresponding to the cutoff value *α *is approximated by

(3)

where *β*_*i*_(*α*) = *P*(*p*_*i *_≤ *α*) is the marginal power of a single *α*-test applied to gene *i *∈ ℳ_1 _and  denotes the expected number of true rejections when we reject *H*_*i *_for *p*_*i *_<*α*, see Jung [6].

Now, we derive *β*_*i*_(*α*) for gene *i *∈ ℳ_1_. By the standard blocked one-way ANOVA theory under the normality assumption for *ε*_*ijk*_,



and



are independent, where  is the noncentral *χ*^2^-distribution with *ν *degrees of freedom and noncentrality parameter *η*, and . Hence, for the *F*-test statistic (2), we have



where  is the noncentral *F*-distribution with *ν*_1 _and *ν*_2 _degrees of freedom, and noncentrality parameter *η*. Note that, for *i *∈ ℳ_0_,  and *F*_*i *_~*F*_(*K*-1),(*K*-1)(*n*-1)_(0) = *F*_(*K*-1),(*K*-1)(*n*-1)_, the central *F*-distribution.

The marginal powers are expressed as

(4)

where  denotes the 100(1 - *α*) percentile of  distribution. The marginal powers can be calculated using R, SAS or some other packages. Suppose we want *r*_1 _true rejections while controlling the FDR at q*. By combining this with (3) and (4), we obtain two equations

(5)

and

(6)

Note that *r*_1_/*m*_1 _denotes the probability of true rejection. At the design stage of a study, *m *is given by the number of genes included in the chips to be used for microarray experiment, *m*_1 _and {, *i *∈ ℳ_1_, 1 ≤ *k *≤ *K*} are projected based on biological knowledge or estimated from pilot data, and *K*, *r*_1 _(or *r*_1_/*m*_1_) and q* are prespecified. The only unknown variables in (5) and (6) are *α *and *n*. By solving (6) with respect to *α*, we obtain α* = *r*_1 _q*/{*m*_0 _(1 - q*)} and, by plugging this in (5), we obtain an equation for *r*_1 _depending only on *n*,

(7)

**Figure 1:**
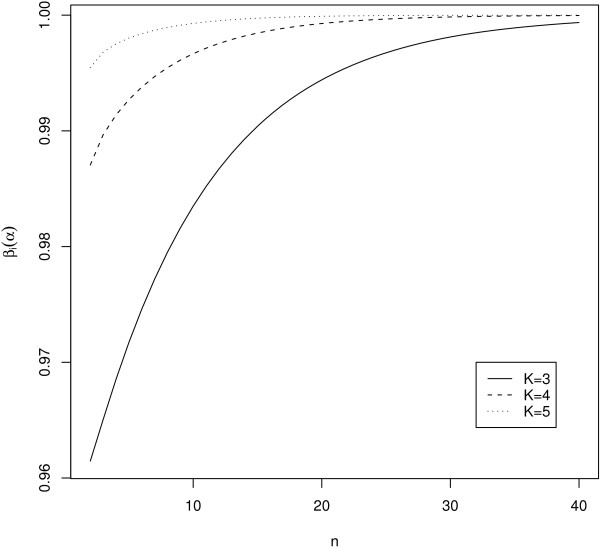
**Monotone relationship between *n *and marginal power *β*_*i *_(*α*) with *α *= 0.05 and  = {*k *- (*K *+ 2)/2}/*K *for 1 ≤ *k *≤ *K***.

The marginal power function (4) includes *n *in the degrees of freedom of the denominator as well as the noncentrality parameter of the *F*-distributions. The impact of the degrees of freedom of the denominator of the *F*-statistic on the marginal power is much weaker than that of the noncentrality parameter, so that *β*_*i*_(*α*) is a monotone increasing function of *n*, and consequently equation (7) has a unique solution. Figure 1 demonstrates the relationship between *n *and *β*_*i*_(*α*) with *α *= 0.05;  = {*k *- (*K *+ 1)/2}/*K *for 1 ≤ *k *≤ *K*; *K *= 3, 4 or 5. This monotone relationship becomes clear for large *n *as shown by an approximate sample size formula given below. Note that the variance of block effect *v*_*i *_has no impact on the sample size and power of the test statistic for treatment effect.

In summary, the sample size (i.e., number of blocks) *n *for *r*_1 _(≤ *m*_1_) true rejections is calculated as follows, assuming that the error terms in model (1) are normally distributed.

#### Sample size calculation based on the noncentral *F*-distribution

(i) Specify the input variables:

- *K *= number of treatments;

- *m *= total number of genes for testing;

- *m*_1 _= number of genes differentially expressed in *K *treatments (*m*_0 _= *m *- *m*_1_);

- {, *i *∈ ℳ_1_, 1 ≤ *k *≤ *K*} = standardized effect sizes for prognostic genes;

- q* = FDR level;

- *r*_1 _= number of true rejections

(ii) Using the bisection method, solve



with respect to *n*, where α* = *r*_1_q*/{*m*_0_(1 - q*)}.

(iii) The required sample size is *n *blocks, or *nK *array chips.

In the sample size formula based on the noncentral *F*-distribution, the relationship between *n *and the marginal power functions based on the *F*-distribution is complicated and a normal distribution assumption of the error terms is required. In the large sample case, we can loosen the normality assumption and simplify this relationship. If the error terms have a finite 4-th moment, then, for large *n*, the distribution of *F*_*i *_is approximated by



A proof is given in the Appendix. Similarly, for large *n*, the *F*_(*K*-1),(*K*-1)(*n*-1) _distribution can be approximated by (*K *- 1)^-1 ^, so that *F*_(*K*-1),(*K*-1)(*n*-1),*α *_≈ (*K *- 1)^-1 ^, where  is the 100(1 - *α*) percentile of the *χ*^2 ^distribution with *ν *degrees of freedom. Hence, the marginal power for *F*_*i *_is approximated by



and a sample size based on the *χ*^2^-distribution approximation is obtained by solving

(8)

with respect to *n*, where α* = *r*_1_q*/{*m*_0_(1 - q*)}. In this equation, *n *appears only in the noncentrality parameter of the *χ*^2 ^distributions.

Equation (8) is especially useful when we want to compare the powers between a blocked one-way design and an unblocked one-way design. Using similar approximations, it is easy to show that an approximate sample size *N *= *nK *for a study with unblocked one-way design with a balanced allocation is obtained by solving

(9)

with respect to *n*, where . The only difference between (8) and (9) is the standardized effect sizes,  = *δ*_*ik*_/*σ *_*i *_and . The latter is always smaller than the former because of the variance among blocks, *v*_*i*_. If *v*_*i *_is large compared to the variance of experimental errors, , then a blocked one-way design requires much smaller number of arrays than an unblocked one-way design. Let *n*_*u *_and *n*_*b *_denote the sample sizes *n *calculated under an unblocked and a blocked design, respectively. If  are constant *f *among the prognostic genes, then from (8) and (9), we have *n*_*u *_= (1 + *f*)*n*_*b*_. As an example, consider the design of the fibrosis study as discussed in Background Section and suppose that the variance of the block effects is half of that of measurement errors for the prognostic genes, i.e. *f *= 0.5. In this case, if a blocked design requires *n*_*b *_= 100 patients and 3*n*_*b *_= 300 array chips, then the corresponding unblocked design with a balanced allocation requires *n*_*u *_= 150 patients per group or a total 450 patients. For an unblocked design, the number of array chips is identical to that of patients, and compared to the blocked design, the unblocked design requires 1.5 times more chips and 4.5 times more patients.

## Results and discussion

### Simulations

First, we investigate the accuracy of the FDR control based on blocked one-way ANOVA tests and the sample size formulas via simulations. For the simulations on FDR control, we consider blocked one-way designs with *K *= 3 treatments and *n *= 10, 30, or 50 blocks. For gene *i *(= 1,..., *m*) from treatment *k *(= 1,..., *K*) of block *j *(= 1,..., *n*), block effect *γ*_*ij *_and error terms *ϵ*_*ijk *_are generated from *N *(0, 0.5^2^) and *N*(0,1), respectively. For differentially expressed genes *i *∈ ℳ_1_, the standardized treatment effects are set at  = (1, 0, -1) or (1, -2, 1). We set the total number of genes *m *= 4000; the number of differentially expressed genes *m*_1 _= 40 or 200; and the nominal FDR level q* = 0.05, 0.1, 0.2, 0.3, 0.4, or 0.5. We conducted *N *= 1000 simulations under each setting, and the null distribution of the test statistics is approximated from *B *= 1000 permutations for each simulation sample. In simulation *l*(= 1,..., *N*), the FDR-control multiple testing method is applied to the simulated data using tuning parameter *λ *= 0.95 [12] to count the numbers of total rejections  and false rejections  and to estimate the FDR, . Then the empirical FDR is obtained as



Table 1 reports the simulation results. The testing procedure controls the FDR accurately, i.e.  ≈ q*, when *m*_1 _is large (*m*_1 _= 200), but tends to be anti-conservative, i.e.  > q*, when *m*_1 _is small (*m*_*i *_= 40). Jung and Jang [13] made similar observations for two-sample t-tests and Cox regression.

**Table 1:** Empirical FDR from N = 1000 simulations with *B *= 1000 permutations for each simulation data set

			*n*		
			
*m*_1_		q*	10	30	50
40	(1, 0, -1)	0.05	0.1766	0.0921	0.0925
		0.1	0.1819	0.1647	0.1705
		0.2	0.2736	0.2462	0.2506
		0.3	0.3636	0.3478	0.3512
		0.4	0.4546	0.4449	0.4431
		0.5	0.5435	0.5389	0.5399
	(1, -2, 1)	0.05	0.0936	0.0899	0.0915
		0.1	0.1619	0.1663	0.1665
		0.2	0.2402	0.2498	0.2421
		0.3	0.3373	0.3469	0.3461
		0.4	0.4347	0.4481	0.4421
		0.5	0.5318	0.5446	0.5340
200	(1, 0, -1)	0.05	0.0653	0.0573	0.0603
		0.1	0.1120	0.1093	0.1130
		0.2	0.2076	0.2105	0.2146
		0.3	0.3079	0.3086	0.3176
		0.4	0.4070	0.4056	0.4171
		0.5	0.5051	0.5013	0.5162
	(1, -2, 1)	0.05	0.0567	0.0554	0.0591
		0.1	0.1108	0.1079	0.1111
		0.2	0.2142	0.2061	0.2116
		0.3	0.3120	0.3052	0.3113
		0.4	0.4124	0.4049	0.4148
		0.5	0.5141	0.5010	0.5162

For the simulations on sample size calculation, we set *m *= 4000; *m*_1 _= 40 or 200; number of treatment *K *= 3; treatment effects  = (1/4, 0, -1/4) or (1/4, -1/2, 1/4) for *i *∈ ℳ_1_; *γ*_*ij *_~*N *(0, 0.5^2^) and *ϵ*_*ijk *_~*N *(0. 1). We want the number of true rejections *r*_1 _to be 30%, 60% or 90% of *m*_1 _while controlling the FDR level at q* = 1%, 5% or 10%. For each design setting, we first calculate the sample size *n *based on the *F*-distribution or the chi-square approximation, and then generate *N *= 1000 samples of size *n *under the same setting. From each simulation sample, the number of true rejections are counted while controlling the FDR at the specified level using *λ *= 0.95. The first, second and third quartiles, *Q*_1_, *Q*_2 _and *Q*_3_, of the observed true rejections, , are estimated from the 1000 simulation samples.

Table 2 summarizes the simulation results by the two methods. As expected, sample size increases in *r*_1 _and decreases in *m*_1 _and q*. Since the standardized effect sizes for the differentially expressed genes influence the sample size through their sum of squares, the combination of effect sizes (1/4, 0, -1/4) requires a larger sample size than (1/4, -1/2, 1/4). The sample size based on the chi-square approximation is always smaller than that based on the *F*-distribution. The median (*Q*_2_) of the empirical true rejections  is smaller than the nominal *r*_1 _for the sample size based on the chi-square approximation, especially with a small *n*, while the sample size based on the *F*-distribution is always accurately powered, i.e. *Q*_2 _≈ *r*_1_.

**Table 2:** *Q*_2 _(*Q*_1_, *Q*_3_)/*n*, where *n *is the sample size and *Q*_*k *_(*k *= 1, 2, 3) are the *k*-th quartile of the empirical true rejections  from *N *= 1000 simulations

		Based on the chi-square approximation		
		
*m*_1_	*r*_1_	q* = 1%	5%	10%
= (1/4, 0, -1/4)				
40	12	11 (9, 13)/123	10 (8, 13)/100	11 (8, 14)/90
	24	23 (20, 26)/166	23 (21, 26)/138	23 (21, 25)/125
	36	36 (34, 37)/242	36 (34, 37)/207	36 (35, 37)/191
200	60	56 (49, 61)/100	55 (47, 61)/77	55 (49, 61)/67
	120	115 (109, 120)/138	118 (112, 124)/110	117 (110, 122)/96
	180	179 (176, 182)/207	178 (176, 182)/171	179 (175, 182)/154
= (1/4, -1/2, 1/4)				
40	12	8 (6, 10)/41	8 (5, 10)/34	7 (5, 10)/30
	24	21 (19, 23)/56	21 (18, 24)/46	21 (19, 24)/42
	36	35 (33, 37)/81	35 (34, 36)/70	36 (34, 37)/64
200	60	42 (36, 48)/34	41 (35, 47)/26	44 (36, 52)/23
	120	103 (98, 109)/46	108 (101, 114)/37	104 (98, 111)/32
	180	176 (173, 180)/70	177 (173, 180)/57	178 (174, 180)/52

		Based on the *F*-distribution		
		
*m*_1_	*r*_1_	q* = 1%	5%	10%

= (1/4, 0, -1/4)				
40	12	12 (10, 15)/129	12 (10, 14)/104	12 (10, 15)/94
	24	24 (21, 27)/171	25 (23, 27)/142	24 (22, 26)/129
	36	36 (35, 37)/246	36 (35, 38)/211	36 (35, 38)/194
200	60	60 (55, 66)/104	61 (54, 66)/80	62 (54, 70)/70
	120	123 (117, 128)/142	122 (118, 128)/113	120 (114, 126)/99
	180	179 (177, 184)/211	180 (177, 183)/174	181 (178, 184)/157
= (1/4, -1/2, 1/4)				
40	12	13 (10, 15)/47	13 (10, 15)/38	13 (10, 16)/34
	24	23 (21, 26)/60	25 (23, 27)/50	25 (23, 27)/46
	36	36 (35, 37)/86	35 (35, 38)/73	36 (34, 37)/67
200	60	61 (55, 67)/38	66 (60, 72)/30	66 (59, 71)/26
	120	121 (116, 127)/50	123 (116, 128)/40	121 (116, 126)/35
	180	180 (177, 183)/73	181 (177, 184)/60	182 (178, 185)/55

### Example

We applied the permutation-based blocked one-way ANOVA and the sample size calculation method to the fibroid study discussed in the Background Section. From each patient, specimens are taken from two sites of fibroid tissue, center (C) and edge (E), and one normal myometrium (M). Five patients are accrued to the study. We regard the three sites as treatments (*K *= 3) and the patients as blocks (*n *= 5). mRNA was amplified and hybridized onto HG-U133 GeneChips according to the protocols recommended by Affymetrix (Santa Clara, CA), and *m *= 54675 probe sets on the array were analyzed. Expression values were calculated using the Robust Multichip Average (RMA) method [14]. RMA estimates are based upon a robust average of background corrected PM intensities. Normalization was done using quantile normalization [15]. We filtered out all "AFFX" genes and genes for which there were 4 or fewer present calls (based on Affymetrix's present/marginal/absent (PMA) calls using mismatch probe intensity, the ratio of PM to MM). That is, a gene is included only if there are at least 3 present calls among the 15 PMA calls. Filtering yielded 30711 genes to be used in the subsequent analyses.

**Figure 2:**
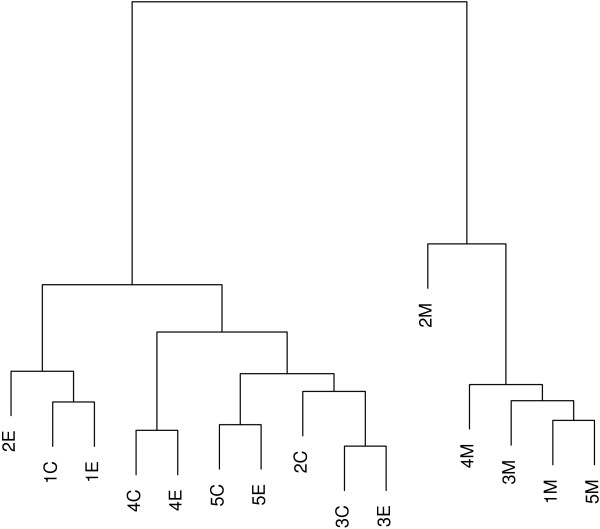
**Hierarchical clustering dendrogram**. *k*A means site A (= E, C or M) for patient *k *(= 1,..., 5).

**Figure 3:**
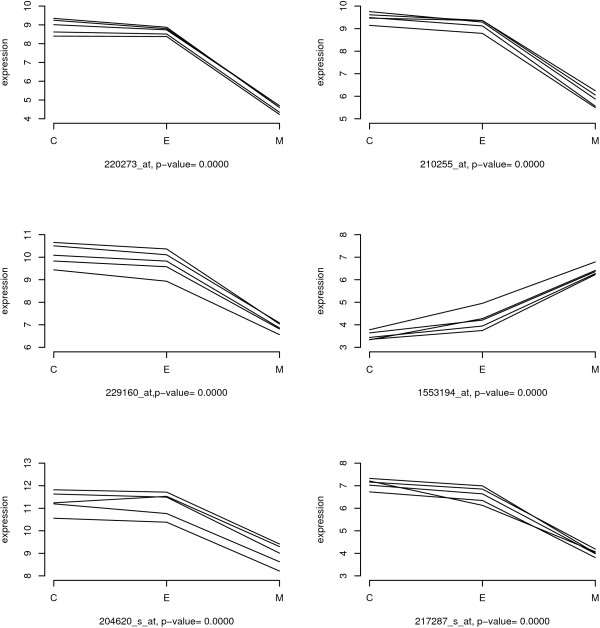
**Expression patterns of six genes that are significantly differentially expressed in three sites**.

In order to group the samples according to the degree of similarity present in the gene expression data, we first applied a hierarchical clustering analysis to the filtered 30711 gene expression data and generated a dendrogram (Figure 2). We used the Complete Linkage method [16] and Pearson's correlation coefficient as a measure of similarity. In the dendrogram, the height of each branch point indicates the similarity level at which each cluster was generated. We obtained the same clustering using the *L*_2 _norm as a measure of similarity. Except for patient 2, E and C are clustered together for each patient. In spite of the block effect, M is clustered separately from E and C regardless of patient assignment. We conclude that C and E have similar gene expression profiles, but M has a different gene expression profile from either C or E. While the clustering analysis investigates the genome wide expression profile, blocked one-way ANOVA helps us identify individual genes differentially expressed among the three sites. Using the blocked one-way ANOVA method, we selected the top 50 genes in terms of parametric p-values (Table 3). The expression patterns of six genes that are identified as differentially expressed are presented in Figure 3. The expression levels of each patients are connected among three sites. These genes are similarly expressed between C and E, but differentially expressed in M. Further, 220273_*at*, 210255_*at*, 229160_*at*, 204620_*s*_*at *and 217287_*s*_*at *are under-expressed in M while 1553194_*at *is over-expressed in M.

**Table 3:** The result of unterine fibroid tissue and adjacent myometrium microarray experiment

		parametric		non-parametric	
		
*probe_set_id*	*Gene_Descriptor*	p-value	q-value	p-value	q-value
220273_*at*	interleukin 17B	0.0000	0.0000	0.0008	0.0131
213479_*at*	neuronal pentraxin II	0.0000	0.0000	0.0015	0.0144
210255_*at*	RAD51-like 1 (S. cerevisiae)	0.0000	0.0000	0.0008	0.0131
205833_*s*_*at*	prostate androgen-regulated transcript 1	0.0000	0.0000	0.0077	0.0219
229160_*at*	melanoma associated antigen (mutated) 1-like 1	0.0000	0.0000	0.0008	0.0131
1561122_*a*_*at*	RAD51-like 1 (S. cerevisiae)	0.0000	0.0000	0.0046	0.0189
210817_*s*_*at*	calcium binding and coiled-coil domain 2	0.0000	0.0000	0.0015	0.0144
1553194_*at*	neuronal growth regulator 1	0.0000	0.0000	0.0008	0.0131
202965_*s*_*at*	calpain 6	0.0000	0.0000	0.0108	0.0239
204620_*s*_*at*	chondroitin sulfate proteoglycan 2 (versican)	0.0000	0.0000	0.0054	0.0196
217287_*s*_*at*	transient receptor potential cation channel, subfamily C, member 6	0.0000	0.0000	0.0008	0.0131
227875_*at*	kelch-like 13 (Drosophila)	0.0000	0.0000	0.0023	0.0156
205286_*at*	transcription factor AP-2 gamma (activating enhancer binding protein 2 gamma)	0.0000	0.0000	0.0046	0.0189
242737_*at*	RAD51-like 1 (S. cerevisiae)	0.0000	0.0000	0.0062	0.0206
209965_*s*_*at*	RAD51-like 3 (S. cerevisiae)	0.0000	0.0000	0.0008	0.0131
202007_*at*	nidogen 1	0.0000	0.0000	0.0015	0.0144
221731_*x*_*at*	chondroitin sulfate proteoglycan 2 (versican)	0.0000	0.0000	0.0077	0.0219
244813_*at*	RAD51-like 1 (S. cerevisiae)	0.0000	0.0000	0.0015	0.0144
201310_*s*_*at*	chromosome 5 open reading frame 13	0.0000	0.0000	0.0008	0.0131
210258_*at*	regulator of G-protein signalling 13	0.0000	0.0000	0.0008	0.0131
202589_*at*	thymidylate synthetase	0.0000	0.0000	0.0054	0.0196
228766_*at*	gb:AW299226	0.0000	0.0000	0.0054	0.0196
218380_*at*	NLR family, pyrin domain containing 1	0.0000	0.0000	0.0008	0.0131
201417_*at*	SRY (sex determining region Y)-box 4	0.0000	0.0000	0.0015	0.0144
215972_*at*	Prostate androgen-regulated transcript 1	0.0000	0.0000	0.0093	0.0231
212942_*s*_*at*	KIAA1199	0.0000	0.0000	0.0046	0.0189
202966_*at*	calpain 6	0.0000	0.0000	0.0108	0.0239
205943_*at*	tryptophan 2,3-dioxygenase	0.0000	0.0000	0.0015	0.0144
213668_*s*_*at*	SRY (sex determining region Y)-box 4	0.0000	0.0000	0.0015	0.0144
219454_*at*	EGF-like-domain, multiple 6	0.0000	0.0000	0.0008	0.0131
235503_*at*	ankyrin repeat and SOCS box-containing 5	0.0000	0.0000	0.0069	0.0212
222834_*s*_*at*	guanine nucleotide binding protein (G protein), gamma 12	0.0000	0.0000	0.0008	0.0131
210198_*s*_*at*	proteolipid protein 1 (Pelizaeus-Merzbacher disease, spastic paraplegia 2, uncomplicated)	0.0000	0.0000	0.0015	0.0144
220565_*at*	chemokine (C-C motif) receptor 10	0.0000	0.0000	0.0008	0.0131
237671_*at*	RAD51-like 1 (S. cerevisiae)	0.0000	0.0000	0.0093	0.0231
201220_*x*_*at*	C-terminal binding protein 2	0.0000	0.0000	0.0039	0.0180
217771_*at*	golgi phosphoprotein 2	0.0000	0.0000	0.0015	0.0144
224002_*s*_*at*	FK506 binding protein 7	0.0000	0.0000	0.0008	0.0131
213170_*at*	glutathione peroxidase 7	0.0000	0.0000	0.0008	0.0131
211980_*at*	collagen, type IV, alpha 1	0.0000	0.0000	0.0031	0.0167
211981_*at*	collagen, type IV, alpha 1	0.0000	0.0000	0.0031	0.0167
212282_*at*	transmembrane protein 97	0.0000	0.0000	0.0008	0.0131
2013090_*x*_*at*	chromosome 5 open reading frame 13	0.0000	0.0000	0.0015	0.0144
211917_*s*_*at*	prolactin receptor///prolactin receptor	0.0000	0.0000	0.0008	0.0131
212281_*s*_*at*	transmembrane protein 97	0.0000	0.0001	0.0008	0.0131
231930_*at*	ELMO/CED-12 domain containing 1	0.0000	0.0001	0.0123	0.0248
205347_*s*_*at*	thymosin-like 8	0.0000	0.0001	0.0015	0.0144
223571_*at*	C1q and tumor necrosis factor related protein 6	0.0000	0.0001	0.0015	0.0144
204619_*s*_*at*	chondroitin sulfate proteoglycan 2 (versican)	0.0000	0.0001	0.0046	0.0189
231741_*at*	endothelial differentiation, sphingolipid G-protein-coupled receptor, 3	0.0000	0.0001	0.0054	0.0196

The results of our analysis of the two sites of fibroid tissue, center and edge, compared to the normal myometrium using a blocked one-way design suggest that reduced FDR provides an enhanced approach to clinical microarray studies. Our findings are consistent with previously reported genome-wide profiling studies [17,18]. We believe that these results support the hypothesis that uterine fibroids develop through altered wound healing signaling pathways leading to tissue fibrosis [19,20]. Using the method described in this paper, genes differentially over-expressed in the fibroid tissue compared to myometrium are related to extracellular matrix (ECM) and ECM regulation such as collagen IV, alpha 1, versican (chondroitin sulfated 2) and IL-17*β *[21]. IL-17*β*, a cell-cell signaling transducer has been reported to enhance MMP secretion and to rapidly induce phosphorylation of the extracellular signal-related kinases (ERK) 1/2 and p38MAPK in colonic myofibroblasts and has been shown to stimulate MMP-1 expression in cardiac fibroblasts through ERK 1/2 and p38 MAPK [22,23]. Thus IL-17*β *is important in remodelling of the extracellular matrix. According to our analysis, RAD51-like 1, a recombinational repair gene, is also over-expressed in fibroids, which is consistent with a report that RAD51B is the preferential translocation partner of high mobility group protein gene (HMGIC) in uterine leiomyomas [24]. HMGIC codes for a protein that is a non-histone DNA binding factor that is expressed during development in embryonic tissue and is an important regulator of cell growth, differentiation and transformation as well as apoptosis [25]. Arrest of apoptosis appears to be a hallmark of uterine fibroids, a finding that is characteristic of altered wound healing as well [19]. HMGIC appears to play a role in the development of uterine fibroids [19,26,27].

Suppose that we want to design a new fibroid study using the data analyzed above as pilot data. In the sample size calculation, we set *m *= 30, 000. We assume that the *m*_1 _= 50 genes which were selected as the top 50 genes in terms of parametric p-value are differentially expressed in the three sites (*K *= 3). From the pilot data, we estimate the standardized treatment effect *δ*_*ik*_. For illustration, the effect sizes of these *m*_1 _= 50 genes are taken to be *δ*_*ik *_= 0.1 . We need *n *= 15 patients (blocks) to discover 90% of the prognostic genes, i.e. *r*_1 _= [0.9 × 50] = 45, while controlling the FDR at q* = 5% level. In a simulation study, we generated *N *= 1000 microarray data sets of size *n *= 15 under this design setting. With q* = 0.05, we observed the quartiles *Q*_2_(*Q*_1_, *Q*_3_) = 46(45, 47) from the empirical distribution of the observed true rejections.

## Conclusion

We have considered studies where microarray data for *K *treatment groups are collected from the same subjects (blocks). We discover the genes differentially expressed among *K *groups using non-parametric *F*-statistics for blocked one-way ANOVA while controlling the FDR. We employ a permutation method to generate the null distribution of the *F*-statistics without a normal distribution assumption for the gene expression data. The permutation-based multiple testing procedure can be easily modified for controlling the familywise error rate, see e.g. Westfall and Young [28] and Jung *et al*. [29].

We propose a simple sample size calculation method to estimate the required number of subjects (blocks) given the total number of genes *m*, number of differentially expressed genes *m*_1 _and their standardized effect sizes (, 1 ≤ *i *≤ *m*_1_, 1 ≤ *k *≤ *K*) and the number of true rejections *r*_1 _at a specified FDR level q*. Through simulations and analysis of a real data set, we found that the permutation-based analysis method controls the FDR accurately and the sample size formula performs accurately. While we specify the individual effect sizes for the prognostic genes, some investigators [30,31] use a mixture model for the marginal p-values by specifying a distribution for the effect sizes among *m *genes.

Glueck *et al*. [32] propose an exact calculation of average power for the Benjamini-Hochberg [2] procedure for controlling the FDR. Their formula may is useful for deriving sample sizes when the test statistics are independent and the number of hypotheses *m *is small. However, it is not appropriate for designing a microarray study with a large number of dependent test statistics.

A sample size calculation program in R is available from .

## Appendix

We want to prove that *F*_*i *_converges to  in distribution regardless of the normal distribution assumption on *ϵ*_*ijk *_and *γ*_*ij*_. We only assume that . The following is one of key lemmas used to derive the distribution of the *F*-statistics in the standard ANOVA theory, see e.g. Section 3b.4 of Rao [33].

Lemma: Suppose that, for *k *= 1,..., *K*, *z*_*k *_are independent *N *(*μ*_*k*_, 1) random variables and *A *is an idempotent *K *× *K *matrix with rank ***ν***. Let ***z ***= (*z*_1_,..., *z*_*K*_)^*T *^and ***μ ***= (*μ*_1_,..., *μ*_*K*_)^*T*^. Then, .

We have



where  and . By the strong law of large numbers, we have ,  and  almost surely (a.s.).

Hence,



Let  and . Then, *z*_1_,..., *z*_*K *_are independent and, by the central limit theorem, *z*_*k *_is approximately . Let *I *be the *K *× *K *identity matrix, **1 **= (1,..., 1)^*T *^the *K *× 1 vector with components 1, ***z ***= (*z*_1_,..., *z*_*K*_)^*T *^*A *= *I *- *K*^-1 ^**11**^*T*^. Note that *A *is an idempotent matrix with rank *K *- 1 and , where . Then,  is approximately distributed as  by the lemma. Since ,  is approximately distributed as . By combining this result with (A.1) using the Slutsky's theorem, we complete the proof.

## Authors' contributions

SJ proposed the research project and wrote the manuscript. IS performed statistical analysis. SLG supported the research and participated in the writing of the manuscripted. PCL was responsible for the study design, conduct and oversight of the experiments and interpretation of results. She contributed to the preparation of the manuscript. LF was responsible for preparing the tissue samples for microarray analysis and interpretation of results and in manuscript preparation. The authors are solely responsible for the content of this study. All authors read and approved the final manuscript.
